# (2*Z*,2′*Z*,4*E*,4′*E*)-4,4′-(Cyclo­hexane-1,2-diyldinitrilo)dipent-2-en-2-ol

**DOI:** 10.1107/S160053680800977X

**Published:** 2008-04-16

**Authors:** Xiu-Zhi Li, Zhi-Rong Qu

**Affiliations:** a Ordered Matter Science Research Center, College of Chemistry and Chemical Engineering, Southeast University, Nanjing 210096, People’s Republic of China

## Abstract

A new tetra­dentate chiral Schiff base ligand, C_16_H_26_N_2_O_2_, has been synthesized by the reaction of acetyl­acetone with (1*R*,2*R*)-(−)-1,2-diamino­cyclo­hexane. Both of the mol­ecules in the asymmetric unit are of the same chirality (*R* configuration), since the absolute configuration was determined by the starting reagent (1*R*,2*R*)-(−)-1,2-diamino­cyclo­hexane. The six-membered cyclo­hexane ring is in a chair conformation, and the substituents are equatorial in the most stable conformation (*trans*-cyclo­hexyl). At the ring substituents, large conjugated —C=N—CH=C—OH systems exist, resulting from the original ketone converted into the enol form. With H atoms excluded, the atoms of each substituent lie in the same plane. The two mol­ecules in the asymmetric unit have almost the same structure, with slight differences in the torsion angles between the substituents and the cyclo­hexane ring; the corresponding N^1^—(C—C—C)_cyclo­hexa­ne_ torsion angles are −177.2 (3) and 179.3 (4)° in one mol­ecule and −176.5 (3) and 178.4 (4)° in the other. Two intra­molecular O—H⋯N hydrogen bonds exist in each mol­ecule.

## Related literature

For the chemistry of Schiff bases, see: Alemi & Shaabani (2000 ▶); Bandini *et al.* (1999 ▶, 2000 ▶); Belokon *et al.* (1997 ▶); Cozzi (2003 ▶); Jiang *et al.* (1995 ▶); Kureshy *et al.* (2001 ▶); Sasaki *et al.* (1991 ▶).
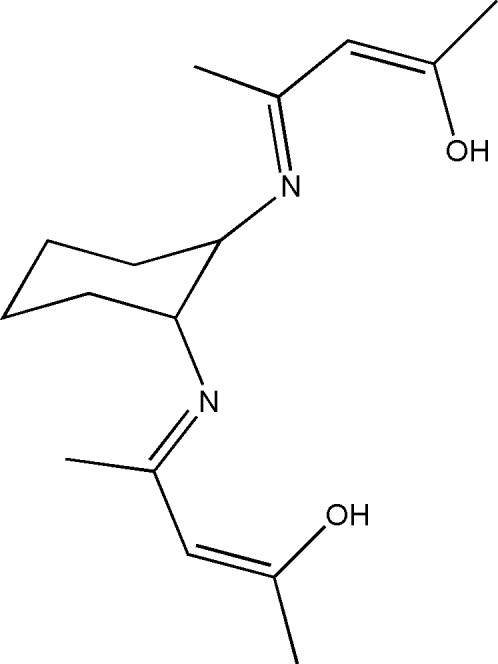


## Experimental

### 

#### Crystal data

C_16_H_26_N_2_O_2_
*M* *_r_* = 278.39
Monoclinic, 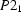

*a* = 9.7306 (15) Å
*b* = 14.7003 (17) Å
*c* = 12.760 (2) Å
β = 109.927 (8)°
*V* = 1716.0 (4) Å^3^
*Z* = 4
Mo *K*α radiation
μ = 0.07 mm^−1^
*T* = 293 (2) K
0.20 × 0.15 × 0.10 mm

#### Data collection

Rigaku SCXmini diffractometer
Absorption correction: multi-scan (*CrystalClear*; Rigaku, 2005 ▶) *T*_min_ = 0.986, *T*_max_ = 0.993
15462 measured reflections
3485 independent reflections
2740 reflections with *I* > 2σ(*I*)
*R*_int_ = 0.046

#### Refinement

*R*[*F* ^2^ > 2σ(*F* ^2^)] = 0.061
*wR*(*F* ^2^) = 0.167
*S* = 1.10
3485 reflections
371 parameters
1 restraint
H-atom parameters constrained
Δρ_max_ = 0.26 e Å^−3^
Δρ_min_ = −0.20 e Å^−3^

### 

Data collection: *CrystalClear* (Rigaku, 2005 ▶); cell refinement: *CrystalClear*; data reduction: *CrystalClear*; program(s) used to solve structure: *SHELXS97* (Sheldrick, 2008 ▶); program(s) used to refine structure: *SHELXL97* (Sheldrick, 2008 ▶); molecular graphics: *SHELXTL* (Sheldrick, 2008 ▶); software used to prepare material for publication: *SHELXTL*.

## Supplementary Material

Crystal structure: contains datablocks I, global. DOI: 10.1107/S160053680800977X/kp2164sup1.cif

Structure factors: contains datablocks I. DOI: 10.1107/S160053680800977X/kp2164Isup2.hkl

Additional supplementary materials:  crystallographic information; 3D view; checkCIF report

## Figures and Tables

**Table 1:** Hydrogen-bond geometry (Å, °)

*D*—H⋯*A*	*D*—H	H⋯*A*	*D*⋯*A*	*D*—H⋯*A*
O4—H4*A*⋯N2	0.85	1.89	2.644 (4)	147
O1—H1*A*⋯N3	0.82	2.00	2.684 (4)	141
O3—H3*A*⋯N1	0.85	1.90	2.662 (5)	148
O2—H2⋯N4	0.82	1.95	2.659 (5)	145

**Fig. 1.**
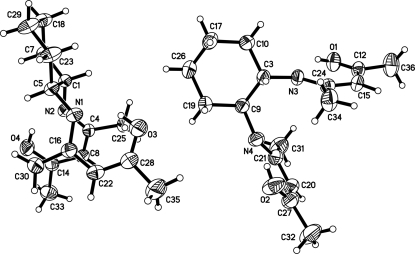
A view of the compound with the atomic numbering scheme. Displacement ellipsoids were drawn at the 30% probability level.

## supplementary crystallographic information

### Comment

In recent years, research on Schiff bases has intensified because some of them form materials with non-linear optical (NLO) activity (Alemi & Shaabani, 2000) and because some can be used for the asymmetric oxidation of methyl phenyl sulfides (Sasaki *et al.*, 1991). The search for new chiral ligands for asymmetric synthesis is an important task in organic chemistry. Various chiral Schiff bases are widely used in asymmetric reactions (Jiang *et al.*, 1995; Belokon *et al.*, 1997; Bandini *et al.*, 1999, 2000; Kureshy *et al.*, 2001; Cozzi, 2003). Herein, we report the synthesis and crystal structure of a new chiral Schiff base ligand (2*Z*,2'*Z*,4E,4'*E*)-4,4'-(cyclohexane-1,2-diylbis(azan-1-yl-1-ylidene))dipent-2-en-2-ol (Fig. 1).

The two molecules in the asymmetric unit have almost the same structure, with slight differences in the torsion angles between the substituents and the cyclohexane ring; the N—C3—C9—C19 and N3—C3—C10—C17 torsion angles are -177.2 (3) and 179.3 (4)°, respectively, and the N2—C1—C5—C23 and N2—C1—C7—C18 torsion angels are -176.5 (3) and 178.4 (4)Å, respectively.

### Experimental

Acetylacetone (2.4 g, 0.024 mol) in 6 ml of chloroform was added dropwise to a solution of chloroform (20 ml) containing (1*R*, 2*R*)-(–)-1,2-Diaminocyclohexane (1.14 g, 0.01 mol), which was kept at 0–5°C with vigorous stirring during the reaction. After complete addition which took approximately 30 min, the mixture was stirred for another 1 h at room temperature. After the evaporation of the solvent under reduced pressure, the crude product was recrystallized by slowly evaporating with petroleum ether to yield pale-yellow crystals.

### Refinement

Hydroxy and methyl H atoms were placed in calculated positions with O—H = 0.82 and C—H = 0.96 Å, and torsion angles were refined. Other H atoms were placed in calculated positions with C—H = 0.93 to 0.98 Å. In the absence of significant anomalous scattering effects, Friedel pairs were averaged.

### Crystal data

**Table:**

C_16_H_26_N_2_O_2_	*F*_000_ = 608
*M**_r_* = 278.39	*D*_x_ = 1.078 Mg m^−^^3^
Monoclinic, *P*2_1_	Mo *K*α radiation λ = 0.71073 Å
Hall symbol: P 2yb	Cell parameters from 3495 reflections
*a* = 9.7306 (15) Å	θ = 3.2–27.5º
*b* = 14.7003 (17) Å	µ = 0.07 mm^−^^1^
*c* = 12.760 (2) Å	*T* = 293 (2) K
β = 109.927 (8)º	Block, yellow
*V* = 1716.0 (4) Å^3^	0.20 × 0.15 × 0.10 mm
*Z* = 4	

### Data collection

**Table:**

Rigaku SCXmini diffractometer	3485 independent reflections
Radiation source: fine-focus sealed tube	2740 reflections with *I* > 2σ(*I*)
Monochromator: graphite	*R*_int_ = 0.046
Detector resolution: 13.6612 pixels mm^-1^	θ_max_ = 26.0º
*T* = 293(2) K	θ_min_ = 3.2º
ω scans	*h* = −11→11
Absorption correction: multi-scan(CrystalClear; Rigaku, 2005)	*k* = −17→18
*T*_min_ = 0.986, *T*_max_ = 0.993	*l* = −15→15
15462 measured reflections	

### Refinement

**Table:**

Refinement on *F*^2^	Secondary atom site location: difference Fourier map
Least-squares matrix: full	Hydrogen site location: inferred from neighbouring sites
*R*[*F*^2^ > 2σ(*F*^2^)] = 0.062	H-atom parameters constrained
*wR*(*F*^2^) = 0.167	*w* = 1/[σ^2^(*F*_o_^2^) + (0.0929*P*)^2^ + 0.0509*P*] where *P* = (*F*_o_^2^ + 2*F*_c_^2^)/3
*S* = 1.10	(Δ/σ)_max_ < 0.001
3485 reflections	Δρ_max_ = 0.26 e Å^−^^3^
371 parameters	Δρ_min_ = −0.20 e Å^−^^3^
1 restraint	Extinction correction: SHELXL97 (Sheldrick, 2008)
Primary atom site location: structure-invariant direct methods	Extinction coefficient: 0.044 (14)

### Special details

**Table:**

Geometry. All e.s.d.'s (except the e.s.d. in the dihedral angle between two l.s. planes) are estimated using the full covariance matrix. The cell e.s.d.'s are taken into account individually in the estimation of e.s.d.'s in distances, angles and torsion angles; correlations between e.s.d.'s in cell parameters are only used when they are defined by crystal symmetry. An approximate (isotropic) treatment of cell e.s.d.'s is used for estimating e.s.d.'s involving l.s. planes.
Refinement. Refinement of *F*^2^ against ALL reflections. The weighted *R*-factor *wR* and goodness of fit *S* are based on *F*^2^, conventional *R*-factors *R* are based on *F*, with *F* set to zero for negative *F*^2^. The threshold expression of *F*^2^ > σ(*F*^2^) is used only for calculating *R*-factors(gt) *etc*. and is not relevant to the choice of reflections for refinement. *R*-factors based on *F*^2^ are statistically about twice as large as those based on *F*, and *R*- factors based on ALL data will be even larger.

### Fractional atomic coordinates and isotropic or equivalent isotropic displacement parameters (Å^2^)

**Table:**

	*x*	*y*	*z*	*U*_iso_*/*U*_eq_	
N2	0.2331 (3)	0.0705 (2)	0.1213 (2)	0.0495 (7)	
C1	0.1320 (3)	0.1372 (3)	0.1382 (3)	0.0506 (8)	
H1	0.1680	0.1981	0.1300	0.061*	
O4	0.2711 (3)	−0.1020 (2)	0.0795 (3)	0.0823 (9)	
H4A	0.2247	−0.0543	0.0851	0.123*	
N1	0.2625 (3)	0.1490 (2)	0.3420 (2)	0.0581 (8)	
N4	0.8253 (4)	0.4606 (3)	0.3268 (3)	0.0678 (9)	
C3	0.6294 (4)	0.5743 (3)	0.2561 (3)	0.0567 (9)	
H3	0.6334	0.5836	0.3331	0.068*	
N3	0.7255 (3)	0.6406 (2)	0.2316 (2)	0.0569 (7)	
C4	0.3731 (4)	0.0815 (2)	0.1303 (3)	0.0495 (8)	
C5	0.1211 (4)	0.1297 (3)	0.2550 (3)	0.0561 (9)	
H5	0.0912	0.0677	0.2654	0.067*	
O1	0.8023 (4)	0.7283 (2)	0.0754 (2)	0.0768 (9)	
H1A	0.7430	0.7005	0.0960	0.115*	
C7	−0.0180 (4)	0.1249 (3)	0.0491 (3)	0.0618 (9)	
H7A	−0.0098	0.1339	−0.0238	0.074*	
H7B	−0.0512	0.0631	0.0524	0.074*	
C8	0.4543 (4)	0.0087 (3)	0.1148 (3)	0.0579 (9)	
H8	0.5499	0.0198	0.1183	0.069*	
C9	0.6805 (4)	0.4776 (3)	0.2460 (3)	0.0614 (10)	
H9	0.6835	0.4687	0.1707	0.074*	
C10	0.4723 (5)	0.5893 (3)	0.1793 (4)	0.0742 (12)	
H10A	0.4418	0.6503	0.1905	0.089*	
H10B	0.4677	0.5843	0.1024	0.089*	
O3	0.4543 (4)	0.2765 (2)	0.4469 (3)	0.0909 (10)	
H3A	0.3750	0.2546	0.4025	0.136*	
C12	0.8823 (4)	0.7750 (3)	0.1538 (4)	0.0634 (10)	
O2	0.9822 (4)	0.4545 (4)	0.5429 (3)	0.1050 (13)	
H2	0.9091	0.4649	0.4887	0.157*	
C14	0.4014 (4)	−0.0812 (3)	0.0939 (3)	0.0618 (9)	
C15	0.8997 (4)	0.7595 (3)	0.2663 (4)	0.0633 (10)	
H15	0.9686	0.7943	0.3198	0.076*	
C16	0.3627 (4)	0.0888 (3)	0.4013 (3)	0.0582 (9)	
C17	0.3679 (5)	0.5206 (3)	0.2010 (4)	0.0783 (12)	
H17A	0.3646	0.5298	0.2754	0.094*	
H17B	0.2703	0.5303	0.1481	0.094*	
C18	−0.1303 (5)	0.1907 (4)	0.0636 (4)	0.0777 (12)	
H18A	−0.1029	0.2524	0.0524	0.093*	
H18B	−0.2249	0.1781	0.0080	0.093*	
C19	0.5730 (5)	0.4084 (3)	0.2667 (4)	0.0786 (12)	
H19A	0.6023	0.3474	0.2544	0.094*	
H19B	0.5780	0.4126	0.3438	0.094*	
C20	1.0821 (5)	0.4578 (4)	0.4000 (4)	0.0835 (14)	
H20	1.1693	0.4558	0.3852	0.100*	
C21	0.9538 (5)	0.4634 (3)	0.3102 (4)	0.0705 (11)	
C22	0.4950 (5)	0.1197 (3)	0.4755 (4)	0.0684 (11)	
H22	0.5618	0.0764	0.5160	0.082*	
C23	0.0078 (5)	0.1962 (4)	0.2683 (4)	0.0831 (14)	
H23A	−0.0012	0.1878	0.3410	0.100*	
H23B	0.0407	0.2579	0.2643	0.100*	
C24	0.8217 (4)	0.6967 (3)	0.3024 (3)	0.0603 (9)	
C25	0.4427 (4)	0.1743 (3)	0.1572 (4)	0.0683 (11)	
H25A	0.3843	0.2180	0.1049	0.103*	
H25B	0.5390	0.1725	0.1525	0.103*	
H25C	0.4489	0.1914	0.2313	0.103*	
C26	0.4153 (5)	0.4247 (4)	0.1903 (5)	0.0848 (14)	
H26A	0.4074	0.4132	0.1136	0.102*	
H26B	0.3510	0.3825	0.2096	0.102*	
C27	1.0909 (5)	0.4549 (4)	0.5141 (4)	0.0850 (15)	
C28	0.5353 (5)	0.2123 (4)	0.4938 (4)	0.0819 (13)	
C29	−0.1412 (5)	0.1825 (5)	0.1784 (4)	0.0909 (15)	
H29A	−0.1788	0.1229	0.1868	0.109*	
H29B	−0.2091	0.2278	0.1872	0.109*	
C30	0.3316 (5)	−0.0111 (3)	0.3878 (4)	0.0731 (12)	
H30A	0.2400	−0.0236	0.3978	0.110*	
H30B	0.4082	−0.0440	0.4426	0.110*	
H30C	0.3266	−0.0299	0.3145	0.110*	
C31	0.9560 (6)	0.4700 (5)	0.1934 (5)	0.0945 (16)	
H31A	0.8864	0.4281	0.1463	0.142*	
H31B	1.0520	0.4553	0.1930	0.142*	
H31C	0.9312	0.5308	0.1661	0.142*	
C32	1.2405 (6)	0.4531 (6)	0.6027 (5)	0.130 (3)	
H32A	1.2634	0.5123	0.6357	0.195*	
H32B	1.3118	0.4362	0.5696	0.195*	
H32C	1.2414	0.4096	0.6590	0.195*	
C33	0.5043 (6)	−0.1553 (4)	0.0876 (5)	0.0952 (16)	
H33A	0.4571	−0.1936	0.0246	0.143*	
H33B	0.5312	−0.1909	0.1546	0.143*	
H33C	0.5903	−0.1289	0.0795	0.143*	
C34	0.8432 (7)	0.6907 (4)	0.4256 (4)	0.0946 (17)	
H34A	0.7524	0.7036	0.4367	0.142*	
H34B	0.9156	0.7342	0.4658	0.142*	
H34C	0.8752	0.6306	0.4522	0.142*	
C35	0.6841 (7)	0.2341 (6)	0.5773 (8)	0.155 (4)	
H35A	0.7044	0.2976	0.5731	0.232*	
H35B	0.7571	0.1987	0.5608	0.232*	
H35C	0.6853	0.2198	0.6510	0.232*	
C36	0.9633 (7)	0.8527 (5)	0.1258 (5)	0.113 (2)	
H37A	1.0664	0.8447	0.1633	0.169*	
H37B	0.9325	0.9087	0.1493	0.169*	
H37C	0.9430	0.8543	0.0467	0.169*	

### Atomic displacement parameters (Å^2^)

**Table:**

	*U*^11^	*U*^22^	*U*^33^	*U*^12^	*U*^13^	*U*^23^
N2	0.0395 (14)	0.0525 (16)	0.0569 (16)	−0.0021 (12)	0.0168 (11)	−0.0008 (14)
C1	0.0424 (17)	0.0480 (18)	0.0581 (19)	0.0000 (14)	0.0127 (14)	−0.0004 (16)
O4	0.0699 (19)	0.0546 (16)	0.125 (3)	−0.0055 (13)	0.0370 (17)	−0.0164 (17)
N1	0.0479 (16)	0.072 (2)	0.0501 (15)	0.0054 (14)	0.0112 (12)	−0.0002 (15)
N4	0.0515 (19)	0.079 (2)	0.071 (2)	0.0026 (16)	0.0175 (15)	0.0169 (19)
C3	0.058 (2)	0.059 (2)	0.0495 (19)	−0.0100 (17)	0.0134 (15)	0.0080 (18)
N3	0.0557 (17)	0.0589 (18)	0.0532 (16)	−0.0140 (14)	0.0148 (13)	0.0023 (15)
C4	0.0471 (18)	0.0487 (19)	0.0525 (18)	−0.0034 (15)	0.0168 (14)	0.0049 (16)
C5	0.0429 (18)	0.067 (2)	0.054 (2)	0.0012 (16)	0.0109 (15)	0.0006 (18)
O1	0.089 (2)	0.084 (2)	0.0676 (17)	−0.0244 (17)	0.0395 (16)	−0.0090 (16)
C7	0.052 (2)	0.065 (2)	0.061 (2)	0.0033 (17)	0.0101 (16)	−0.0034 (19)
C8	0.0461 (19)	0.060 (2)	0.068 (2)	0.0015 (16)	0.0205 (16)	0.0025 (18)
C9	0.054 (2)	0.065 (2)	0.063 (2)	−0.0031 (17)	0.0166 (17)	0.0076 (19)
C10	0.057 (2)	0.072 (3)	0.088 (3)	−0.001 (2)	0.017 (2)	0.016 (2)
O3	0.079 (2)	0.076 (2)	0.097 (2)	−0.0045 (17)	0.0019 (17)	−0.0026 (19)
C12	0.060 (2)	0.063 (2)	0.076 (3)	−0.0123 (18)	0.035 (2)	−0.008 (2)
O2	0.067 (2)	0.162 (4)	0.081 (2)	0.006 (2)	0.0181 (17)	0.045 (2)
C14	0.059 (2)	0.057 (2)	0.073 (2)	0.0026 (18)	0.0268 (18)	0.000 (2)
C15	0.057 (2)	0.064 (2)	0.065 (2)	−0.0159 (18)	0.0167 (18)	−0.0082 (19)
C16	0.053 (2)	0.071 (2)	0.0521 (19)	0.0056 (18)	0.0207 (16)	0.0084 (19)
C17	0.056 (2)	0.087 (3)	0.088 (3)	−0.012 (2)	0.019 (2)	0.008 (3)
C18	0.053 (2)	0.084 (3)	0.080 (3)	0.014 (2)	0.0019 (19)	−0.008 (2)
C19	0.067 (3)	0.062 (3)	0.103 (3)	−0.009 (2)	0.024 (2)	0.007 (3)
C20	0.055 (3)	0.094 (3)	0.100 (3)	−0.004 (2)	0.024 (2)	0.019 (3)
C21	0.058 (2)	0.071 (3)	0.084 (3)	−0.003 (2)	0.027 (2)	0.012 (2)
C22	0.056 (2)	0.077 (3)	0.064 (2)	0.0063 (19)	0.0089 (18)	0.004 (2)
C23	0.056 (2)	0.118 (4)	0.074 (3)	0.024 (2)	0.019 (2)	−0.012 (3)
C24	0.057 (2)	0.063 (2)	0.055 (2)	−0.0027 (17)	0.0121 (16)	0.0036 (19)
C25	0.054 (2)	0.058 (2)	0.092 (3)	−0.0115 (18)	0.025 (2)	0.000 (2)
C26	0.066 (3)	0.079 (3)	0.100 (4)	−0.020 (2)	0.016 (2)	0.000 (3)
C27	0.064 (3)	0.095 (4)	0.087 (3)	−0.009 (2)	0.013 (2)	0.035 (3)
C28	0.061 (3)	0.085 (3)	0.084 (3)	−0.004 (2)	0.004 (2)	0.005 (3)
C29	0.049 (2)	0.123 (4)	0.099 (3)	0.020 (3)	0.023 (2)	−0.006 (3)
C30	0.074 (3)	0.069 (3)	0.072 (3)	0.001 (2)	0.018 (2)	0.013 (2)
C31	0.078 (3)	0.123 (5)	0.090 (3)	−0.003 (3)	0.040 (3)	−0.001 (3)
C32	0.072 (4)	0.190 (8)	0.105 (4)	−0.031 (4)	0.000 (3)	0.054 (5)
C33	0.088 (3)	0.071 (3)	0.128 (5)	0.018 (3)	0.038 (3)	−0.003 (3)
C34	0.116 (4)	0.101 (4)	0.053 (2)	−0.037 (3)	0.011 (2)	−0.001 (3)
C35	0.096 (5)	0.110 (5)	0.193 (8)	−0.020 (4)	−0.037 (5)	−0.009 (5)
C36	0.125 (5)	0.114 (5)	0.117 (4)	−0.048 (4)	0.066 (4)	−0.001 (4)

### Geometric parameters (Å, °)

**Table:**

N2—C4	1.337 (4)	C18—C29	1.509 (7)
N2—C1	1.456 (4)	C18—H18A	0.9700
C1—C7	1.524 (5)	C18—H18B	0.9700
C1—C5	1.533 (5)	C19—C26	1.531 (6)
C1—H1	0.9800	C19—H19A	0.9700
O4—C14	1.256 (5)	C19—H19B	0.9700
O4—H4A	0.8499	C20—C21	1.379 (6)
N1—C16	1.343 (5)	C20—C27	1.431 (8)
N1—C5	1.471 (5)	C20—H20	0.9300
N4—C21	1.338 (5)	C21—C31	1.502 (7)
N4—C9	1.456 (5)	C22—C28	1.413 (7)
C3—N3	1.457 (5)	C22—H22	0.9300
C3—C10	1.524 (5)	C23—C29	1.525 (6)
C3—C9	1.525 (6)	C23—H23A	0.9700
C3—H3	0.9800	C23—H23B	0.9700
N3—C24	1.340 (5)	C24—C34	1.516 (6)
C4—C8	1.385 (5)	C25—H25A	0.9600
C4—C25	1.509 (5)	C25—H25B	0.9600
C5—C23	1.525 (6)	C25—H25C	0.9600
C5—H5	0.9800	C26—H26A	0.9700
O1—C12	1.244 (5)	C26—H26B	0.9700
O1—H1A	0.8200	C27—C32	1.508 (7)
C7—C18	1.517 (6)	C28—C35	1.511 (7)
C7—H7A	0.9700	C29—H29A	0.9700
C7—H7B	0.9700	C29—H29B	0.9700
C8—C14	1.410 (6)	C30—H30A	0.9600
C8—H8	0.9300	C30—H30B	0.9600
C9—C19	1.544 (6)	C30—H30C	0.9600
C9—H9	0.9800	C31—H31A	0.9600
C10—C17	1.523 (6)	C31—H31B	0.9600
C10—H10A	0.9700	C31—H31C	0.9600
C10—H10B	0.9700	C32—H32A	0.9600
O3—C28	1.245 (6)	C32—H32B	0.9600
O3—H3A	0.8500	C32—H32C	0.9600
C12—C15	1.406 (6)	C33—H33A	0.9600
C12—C36	1.498 (7)	C33—H33B	0.9600
O2—C27	1.232 (6)	C33—H33C	0.9600
O2—H2	0.8200	C34—H34A	0.9600
C14—C33	1.501 (6)	C34—H34B	0.9600
C15—C24	1.371 (6)	C34—H34C	0.9600
C15—H15	0.9300	C35—H35A	0.9600
C16—C22	1.389 (6)	C35—H35B	0.9600
C16—C30	1.498 (6)	C35—H35C	0.9600
C17—C26	1.504 (8)	C36—H37A	0.9600
C17—H17A	0.9700	C36—H37B	0.9600
C17—H17B	0.9700	C36—H37C	0.9600
			
C4—N2—C1	128.7 (3)	C27—C20—H20	117.6
N2—C1—C7	109.5 (3)	N4—C21—C20	119.8 (4)
N2—C1—C5	111.7 (3)	N4—C21—C31	119.3 (4)
C7—C1—C5	110.5 (3)	C20—C21—C31	120.8 (4)
N2—C1—H1	108.4	C16—C22—C28	124.6 (4)
C7—C1—H1	108.4	C16—C22—H22	117.7
C5—C1—H1	108.4	C28—C22—H22	117.7
C14—O4—H4A	109.0	C29—C23—C5	111.7 (4)
C16—N1—C5	127.7 (4)	C29—C23—H23A	109.3
C21—N4—C9	127.8 (4)	C5—C23—H23A	109.3
N3—C3—C10	110.0 (3)	C29—C23—H23B	109.3
N3—C3—C9	110.7 (3)	C5—C23—H23B	109.3
C10—C3—C9	111.4 (3)	H23A—C23—H23B	107.9
N3—C3—H3	108.2	N3—C24—C15	121.9 (3)
C10—C3—H3	108.2	N3—C24—C34	118.8 (4)
C9—C3—H3	108.2	C15—C24—C34	119.3 (4)
C24—N3—C3	128.2 (3)	C4—C25—H25A	109.5
N2—C4—C8	120.6 (3)	C4—C25—H25B	109.5
N2—C4—C25	119.6 (3)	H25A—C25—H25B	109.5
C8—C4—C25	119.8 (3)	C4—C25—H25C	109.5
N1—C5—C23	108.5 (3)	H25A—C25—H25C	109.5
N1—C5—C1	111.3 (3)	H25B—C25—H25C	109.5
C23—C5—C1	110.7 (3)	C17—C26—C19	111.1 (4)
N1—C5—H5	108.7	C17—C26—H26A	109.4
C23—C5—H5	108.7	C19—C26—H26A	109.4
C1—C5—H5	108.7	C17—C26—H26B	109.4
C12—O1—H1A	109.5	C19—C26—H26B	109.4
C18—C7—C1	112.3 (3)	H26A—C26—H26B	108.0
C18—C7—H7A	109.1	O2—C27—C20	123.0 (4)
C1—C7—H7A	109.1	O2—C27—C32	119.0 (5)
C18—C7—H7B	109.1	C20—C27—C32	118.1 (5)
C1—C7—H7B	109.1	O3—C28—C22	123.8 (4)
H7A—C7—H7B	107.9	O3—C28—C35	118.4 (5)
C4—C8—C14	124.2 (3)	C22—C28—C35	117.8 (5)
C4—C8—H8	117.9	C18—C29—C23	110.8 (4)
C14—C8—H8	117.9	C18—C29—H29A	109.5
N4—C9—C3	111.4 (3)	C23—C29—H29A	109.5
N4—C9—C19	108.3 (3)	C18—C29—H29B	109.5
C3—C9—C19	109.9 (3)	C23—C29—H29B	109.5
N4—C9—H9	109.1	H29A—C29—H29B	108.1
C3—C9—H9	109.1	C16—C30—H30A	109.5
C19—C9—H9	109.1	C16—C30—H30B	109.5
C17—C10—C3	111.9 (4)	H30A—C30—H30B	109.5
C17—C10—H10A	109.2	C16—C30—H30C	109.5
C3—C10—H10A	109.2	H30A—C30—H30C	109.5
C17—C10—H10B	109.2	H30B—C30—H30C	109.5
C3—C10—H10B	109.2	C21—C31—H31A	109.5
H10A—C10—H10B	107.9	C21—C31—H31B	109.5
C28—O3—H3A	108.4	H31A—C31—H31B	109.5
O1—C12—C15	123.8 (4)	C21—C31—H31C	109.5
O1—C12—C36	117.7 (4)	H31A—C31—H31C	109.5
C15—C12—C36	118.5 (4)	H31B—C31—H31C	109.5
C27—O2—H2	109.5	C27—C32—H32A	109.5
O4—C14—C8	122.8 (4)	C27—C32—H32B	109.5
O4—C14—C33	118.3 (4)	H32A—C32—H32B	109.5
C8—C14—C33	118.9 (4)	C27—C32—H32C	109.5
C24—C15—C12	124.3 (4)	H32A—C32—H32C	109.5
C24—C15—H15	117.8	H32B—C32—H32C	109.5
C12—C15—H15	117.8	C14—C33—H33A	109.5
N1—C16—C22	119.6 (4)	C14—C33—H33B	109.5
N1—C16—C30	120.1 (4)	H33A—C33—H33B	109.5
C22—C16—C30	120.2 (4)	C14—C33—H33C	109.5
C26—C17—C10	111.2 (4)	H33A—C33—H33C	109.5
C26—C17—H17A	109.4	H33B—C33—H33C	109.5
C10—C17—H17A	109.4	C24—C34—H34A	109.5
C26—C17—H17B	109.4	C24—C34—H34B	109.5
C10—C17—H17B	109.4	H34A—C34—H34B	109.5
H17A—C17—H17B	108.0	C24—C34—H34C	109.5
C29—C18—C7	111.1 (4)	H34A—C34—H34C	109.5
C29—C18—H18A	109.4	H34B—C34—H34C	109.5
C7—C18—H18A	109.4	C28—C35—H35A	109.5
C29—C18—H18B	109.4	C28—C35—H35B	109.5
C7—C18—H18B	109.4	H35A—C35—H35B	109.5
H18A—C18—H18B	108.0	C28—C35—H35C	109.5
C26—C19—C9	112.3 (4)	H35A—C35—H35C	109.5
C26—C19—H19A	109.1	H35B—C35—H35C	109.5
C9—C19—H19A	109.1	C12—C36—H37A	109.5
C26—C19—H19B	109.1	C12—C36—H37B	109.5
C9—C19—H19B	109.1	H37A—C36—H37B	109.5
H19A—C19—H19B	107.9	C12—C36—H37C	109.5
C21—C20—C27	124.7 (4)	H37A—C36—H37C	109.5
C21—C20—H20	117.6	H37B—C36—H37C	109.5
			
C4—N2—C1—C7	139.4 (4)	C36—C12—C15—C24	173.5 (5)
C4—N2—C1—C5	−97.9 (4)	C5—N1—C16—C22	175.8 (4)
C10—C3—N3—C24	127.5 (4)	C5—N1—C16—C30	−4.5 (6)
C9—C3—N3—C24	−108.9 (4)	C3—C10—C17—C26	−56.1 (6)
C1—N2—C4—C8	177.7 (3)	C1—C7—C18—C29	−55.9 (5)
C1—N2—C4—C25	−2.6 (6)	N4—C9—C19—C26	176.4 (4)
C16—N1—C5—C23	140.5 (4)	C3—C9—C19—C26	54.5 (5)
C16—N1—C5—C1	−97.5 (4)	C9—N4—C21—C20	171.0 (4)
N2—C1—C5—N1	62.6 (4)	C9—N4—C21—C31	−10.7 (7)
C7—C1—C5—N1	−175.3 (3)	C27—C20—C21—N4	−4.3 (8)
N2—C1—C5—C23	−176.5 (3)	C27—C20—C21—C31	177.5 (5)
C7—C1—C5—C23	−54.5 (4)	N1—C16—C22—C28	−0.7 (7)
N2—C1—C7—C18	178.4 (4)	C30—C16—C22—C28	179.7 (5)
C5—C1—C7—C18	55.1 (5)	N1—C5—C23—C29	178.1 (4)
N2—C4—C8—C14	−3.0 (6)	C1—C5—C23—C29	55.7 (5)
C25—C4—C8—C14	177.3 (4)	C3—N3—C24—C15	−176.3 (4)
C21—N4—C9—C3	−98.6 (5)	C3—N3—C24—C34	3.1 (7)
C21—N4—C9—C19	140.5 (5)	C12—C15—C24—N3	3.4 (7)
N3—C3—C9—N4	62.7 (4)	C12—C15—C24—C34	−175.9 (5)
C10—C3—C9—N4	−174.6 (3)	C10—C17—C26—C19	55.1 (6)
N3—C3—C9—C19	−177.2 (3)	C9—C19—C26—C17	−55.2 (6)
C10—C3—C9—C19	−54.5 (4)	C21—C20—C27—O2	2.2 (9)
N3—C3—C10—C17	179.3 (4)	C21—C20—C27—C32	−177.3 (6)
C9—C3—C10—C17	56.1 (5)	C16—C22—C28—O3	1.8 (9)
C4—C8—C14—O4	5.3 (7)	C16—C22—C28—C35	−179.7 (6)
C4—C8—C14—C33	−175.1 (4)	C7—C18—C29—C23	55.6 (6)
O1—C12—C15—C24	−5.8 (7)	C5—C23—C29—C18	−56.3 (6)

### Hydrogen-bond geometry (Å, °)

**Table:**

*D*—H···*A*	*D*—H	H···*A*	*D*···*A*	*D*—H···*A*
O4—H4A···N2	0.85	1.89	2.644 (4)	147
O1—H1A···N3	0.82	2.00	2.684 (4)	141
O3—H3A···N1	0.85	1.90	2.662 (5)	148
O2—H2···N4	0.82	1.95	2.659 (5)	145
